# Diagnostic accuracy of exhaled nitric oxide for the non-invasive identification of patients with fibrotic metabolic dysfunction-associated steatohepatitis

**DOI:** 10.1080/07853890.2024.2410408

**Published:** 2024-10-08

**Authors:** Huai Zhang, Ou-Yang Huang, Li-Li Chen, Ni Zhang, Wen-Ying Chen, Wen Zheng, Xin-Lei Zhang, Xiao-Zhi Jin, Sui-Dan Chen, Giovanni Targher, Christopher D. Byrne, Ming-Hua Zheng

**Affiliations:** a Department of Biostatistics and Medical Record, the First Affiliated Hospital of Wenzhou Medical University, Wenzhou, China; b MAFLD Research Centre, Department of Hepatology, the First Affiliated Hospital of Wenzhou Medical University, Wenzhou, China; c Department of Pathology, the First Affiliated Hospital of Wenzhou Medical University, Wenzhou, China; d Department of Medicine, University of Verona, Verona, Italy; e IRCCS Sacro Cuore Don Calabria Hospital, Negrar di Valpolicella, Italy; f Southampton National Institute for Health and Care Research Biomedical Research Centre, University Hospital Southampton and University of Southampton, Southampton General Hospital, Southampton, UK; g Key Laboratory of Diagnosis and Treatment for the Development of Chronic Liver Disease in Zhejiang Province, Wenzhou, China

**Keywords:** Exhaled nitric oxide, breath test, fibrotic MASH

## Abstract

**Background:**

Fibrotic metabolic dysfunction-associated steatohepatitis (MASH) is a condition at risk of progressing to advanced liver disease. We examined whether an innovative exhaled nitric oxide (eNO) breath test (BT) can accurately diagnose fibrotic MASH without requiring blood tests.

**Methods:**

One hundred and forty-seven patients with MASH were recruited, and all tests were undertaken within 1 week of recruitment. With fibrotic MASH (NAS ≥ 4 and fibrosis stage ≥ 2) as the main outcome indicator, the diagnostic efficacy of eNO in identifying fibrotic MASH was compared to other validated models for advanced fibrosis requiring venesection, namely FAST, Agile 3^+^, and FIB-4 scores.

**Results:**

The mean age was 40.36 ± 12.28 years, 73.5% were men. Mean body mass index was 28.83 ± 4.31 kg/m^2^. The proportion of fibrotic MASH was 29.25%. The area under the receiver operating curve for eNO in diagnosing fibrotic MASH was 0.737 [95% CI 0.650–0.823], which was comparable to FAST (0.751 [0.656–0.846]), Agile 3^+^ (0.764 [0.670–0.858]), and FIB-4 (0.721 [0.620-0.821]) (all DeLong test *p* > 0.05). A cut-off of eNO <8.5 ppb gave a sensitivity of 86.0% and a negative predictive value of 88.5% for ruling-out fibrotic MASH. A cut-off of eNO >13.5 ppb provided a specificity of 91.3% and a positive predictive value of 65.4% for ruling-in fibrotic MASH. Sensitivity analyses demonstrated that the diagnostic efficacy of eNO was similar across characteristics such as age. Moreover, adding vibration-controlled transient elastography-LSM (liver stiffness measurement) reduced the uncertainty interval from 46.9% to 39.5%.

**Conclusions:**

The eNO-BT is a promising simple test for non-invasively identifying fibrotic MASH, and its performance is further improved by adding LSM measurement.

## Introduction

Metabolic dysfunction-associated fatty liver disease (MAFLD), renamed from non-alcoholic fatty liver disease (NAFLD) [1,2], has become the most prevalent form of chronic liver disease worldwide, imposing a significant societal burden [3,4]. MAFLD affects approximately 25%–40% of the global adult population, and the prevalence of MAFLD can be as high as 70% in people living with obesity or type 2 diabetes [3,5]. Metabolic dysfunction-associated steatohepatitis (MASH) is a more aggressive form of MAFLD characterized by hepatic inflammation, which can promote the progressive accumulation of parenchyma fibrosis [6,7]. Over time, if left untreated, MASH can progress to cirrhosis, liver failure, and even hepatocellular carcinoma [8,9], and is associated with an increased risk of liver-related mortality. MASH has become the second leading indication for liver transplantation in the USA and Europe and is expected to become the primary indication in the coming decade [4,10]. Hence, it is important to accurately identify people with fibrotic MASH at high risk of progressing to advanced liver disease.

Non-invasive liver tests represent an appealing alternative to potentially unnecessary, invasive, and costly liver biopsies [11]. Currently, non-invasive tests are mainly serum biomarkers, imaging methodologies, and comprehensive algorithms. However, most of these tests require blood testing that is sometimes not possible or is unacceptable to patients [12], or is intended for assessing the probability of advanced liver fibrosis (stage s, a [13] without assessing liver inflammation. Nevertheless, the good performance of exhaled nitric oxide (eNO) concentration in the non-invasive diagnosis of hepatitis [14,15] and cirrhosis [16,17], along with its correlation with the inflammatory response, can provide a promising avenue for the realization of a truly non-invasive diagnosis for fibrotic MASH. Studies had shown that inducible nitric oxide synthase expression is up-regulated in response to inflammatory factors, which induces the increase of the production of NO with similar activity to oxygen radicals, and in turn aggravates the inflammatory response. The reduced bioavailability of NO by liver sinusoidal endothelial cells is also a key factor in the increase of intrahepatic resistance.

We used fibrotic MASH as the primary outcome, defined by the combined histology criteria of NAFLD Activity Score (NAS) of 4 or higher and fibrosis stage of 2 or higher. This criterion can include patients with fibrotic symptoms and identify subjects who may benefit from future anti-inflammatory therapies. Moreover, these inclusion criteria align with current clinical drug trial standards [18,19].

Therefore, the present study aimed to test for the first time if a truly non-invasive diagnostic test in the form of breath testing could non-invasively identify patients with fibrotic MASH, as opposed to minimally invasive tests that require fasting, venesection, bulky equipment, or other procedures.

## Patients and methods

### Study design

The study population was obtained from the well-characterized Prospective Epidemic Research Specifically of MASH (PERSONS) cohort [20] at the First Affiliated Hospital of Wenzhou Medical University between August 2020 and April 2023. The study protocol was approved by the ethics committee of the First Affiliated Hospital of Wenzhou Medical University (2016-246), and the study was conducted following the tenets of the Declaration of Helsinki published in 1964 and its later amendments, as well as local government policies. All participants signed their written informed consent to participate in the study.

A total of 147 potentially eligible participants who had undergone breath testing and had liver disease were recruited. Participants had radiologic evidence of hepatic steatosis confirmed by subsequent liver histology. Patients were excluded according to the following criteria: (a) subjects who had missing data for eNO and liver biopsy; (b) subjects who were diagnosed with active malignancy or other terminal diseases, or subjects who had participated in another clinical trial within the previous 30 days; and (c) subjects whose steatosis is <5% on liver biopsy.

Age, sex, body mass index (BMI), waist circumference, hip circumference, and waist-to-hip ratio (WHR) were recorded. Disease history, smoking status, and alcohol consumption were recorded by questionnaire. An 8-h fasting blood sample was obtained in the hospital and transferred within half an hour for analyte assessment.

### Procedures

#### Liver biopsy

Under ultrasound guidance, all patients underwent a percutaneous liver biopsy using a 16-gauge Hepafix needle. Specimens were embedded in paraffin and stained with haematoxylin–eosin and Masson. Slides were reviewed by an experienced liver pathologist (Chen SD), blinded to patients’ clinical details. Histological liver characteristics, such as steatosis (grade, 0–3), ballooning (grade, 0–2), lobular inflammation (grade, 0–3) grades, and fibrosis stage (stage, 0–4) were assessed according to the NASH Clinical Research Network scoring system [21]. The histologic NAFLD activity score (NAS ranging from 0 to 8) was calculated as the sum of steatosis, ballooning, and lobular inflammation. The study’s main outcome was the identification of “fibrotic MASH,” defined as NAS of 4 or higher (at least one point for steatosis, ballooning, and lobular inflammation) and fibrosis stage 2 or higher.

#### Vibration-controlled transient elastography measurement

Controlled attenuation parameter (CAP) and liver stiffness measurement (LSM) were assessed by Fibroscan devices equipped with M (Echosens, Paris, France) by nurses or physicians trained and certified by the manufacturer [22,23]. The examinations were conducted with patients fasting for at least 3 h by the above-trained personnel blinded to patients’ data. The patients were supine with the right arm fully abducted, and the right liver lobe was scanned through the intercostal space.

#### Breath test

The concentration of exhaled NO was measured using a Nano Coulomb Breath Analyzer (Sunvou-CA4458, Wuxi, China). The unit of exhaled NO was ppb (parts per billion) (1 ppb = 1 × 10^−9^mol/L). Eating, smoking, drinking, strenuous exercise, or pulmonary function tests were prohibited 1 h before the measurement. During the measurement, the subjects were seated and tightened their lips around a disposable expiratory filter tip. Subjects were informed about inhaling NO-free air and exhaling via the filter tip at flow rates of 200 mL/s. For more measurement details, see the related expert consensus and studies [24,25]. All tests, including breath test (BT), liver elastography, venesection, and liver biopsy, were conducted at intervals of no more than 1 week [26].

### Statistical analysis

Kolmogorov–Smirnov test and Q–Q plot test were used to comprehensively verify the normality of the data. Data were presented as proportions, means ± SD, or medians (inter-quartiles) according to the original data distribution. Correlations were evaluated by Pearson’s or Spearman’s correlation analyses. The diagnostic performance of eNO was assessed in terms of the area under the receiver operating curve (AUROC) and compared with other validated diagnostic models that have good diagnostic performance for identifying advanced liver fibrosis (i.e. Fibroscan-aspartate aminotransferase (FAST) score, Agile 3^+^, and FIB-4) by using the DeLong test [27]. Dual cut-off values were determined based on the optimum balance of sensitivity (Se, ≥0.85) and specificity (Sp, ≥0.90), and that minimized the proportion of the “grey zone.” When appraising performance at a given eNO cut-off value, Se, Sp, positive predictive value (PPV), and negative predictive value (NPV) were computed and graphically presented.

To compensate for the absence of external data for validation, based on the included sample size, 90% of the sample was randomly selected for ROC analysis, and this was repeated 100 times. And consistency analysis was performed on the diagnosis of eNO and FAST in the same population. Statistical analyses were conducted using IBM SPSS statistics (version 26.0) and software R (version 4.2.3). All reported probability values were two-tailed, and a *p*-value <0.05 was considered statistically significant.

## Results

One hundred and forty-seven adult participants with biopsy-confirmed MASH were included in the analysis. The characteristics of these 147 subjects (104 with no fibrotic MASH and 43 with fibrotic MASH) are summarized in Table 1. The mean age was 40.36 ± 12.28 years, 73.5% of the patients were men, and the mean BMI was 28.83 ± 4.31 kg/m^2^. The proportion of fibrotic MASH was 29.25%, and there was a significant increase in exhaled NO content in the participants with fibrotic MASH. Patients with fibrotic MASH were older and were more likely to have type 2 diabetes. In addition, fibrotic MASH participants had significantly higher levels of serum aspartate aminotransferase (AST), alanine aminotransferase (ALT), γ-glutamyl transferase (GGT), LSM, and there were no significant differences in terms of hypertension, respiratory disease, smoking status, and adiposity measures between the two groups.

**Table 1.** Baseline clinical and biochemical characteristics and liver histology features in patients with fibrotic MASH and non-fibrotic MASH.

Characteristics	All (*n* = 147)	Non-fibrotic MASH (*n* = 104)	Fibrotic MASH (*n* = 43)	*p*-value
**Demographics**				
Age (years)	40.36 ± 12.28	38.68 ± 11.64	44.42 ± 12.96	**0.010**
Male sex (n, %)	108 (73.5%)	81 (77.9%)	27 (62.8%)	0.059
BMI (kg/m^2^)	28.83 ± 4.31	28.99 ± 4.43	28.43 ± 4.04	0.477
Waist circumference (cm)	97.03 ± 8.15	97.22 ± 7.81	96.57 ± 9.01	0.668
WHR	0.95 ± 0.05	0.95 ± 0.05	0.96 ± 0.05	0.393
Smoking history (non/former/current; n, %)	88/12/37 (64.2%/8.8%/27.0%)	60/9/28 (61.9%/9.3%/28.9%)	28/3/9 (70.0%/7.5%/22.5%)	0.733
**Concomitant diseases**				
Type 2 diabetes (n, %)	45 (30.6%)	24 (23.1%)	21 (48.8%)	**0.002**
Hypertension (n, %)	31 (21.1%)	19 (18.3%)	12 (27.9%)	0.193
Respiratory disease (n, %)	13 (8.8%)	11 (10.6%)	2 (4.7%)	0.405
**Blood parameters**				
AST (IU/L)	42 (27–67)	37 (25–51)	69 (34–96)	**<0.001**
ALT (IU/L)	65 (36–106)	57 (33–98)	97 (55–120)	**0.007**
GGT (IU/L)	51 (33–96)	48 (27–73)	74 (47–125)	**0.002**
Platelet count (×10^9^/L)	248.29 ± 62.02	255.73 ± 56.29	230.47 ± 71.58	**0.024**
**Fibroscan data**				
LSM (kPa)	7.30 (6.00–9.73)	6.85 (5.40–8.75)	9.90 (7.40–13.55)	**<0.001**
CAP (dB/m)	308.50 ± 44.06	305.89 ± 46.76	317.12 ± 35.79	0.164
**Non-invasive models**				
eNO (ppb)	10.47 ± 3.99	9.43 ± 3.22	12.98 ± 4.58	**<0.001**
FAST	0.44 (0.22–0.66)	0.40 (0.19–0.55)	0.69 (0.40–0.78)	**<0.001**
Agile 3^+^	0.12 (0.05–0.31)	0.09 (0.04–0.19)	0.36 (0.10–0.69)	**<0.001**
FIB-4	0.80 (0.59–1.24)	0.74 (0.54–1.03)	1.39 (0.72–2.29)	**<0.001**
**Histological liver characteristics**				
**Steatosis grade (n, %)**				
1	46 (31.3%)	36 (34.6%)	10 (23.3%)	0.203
2	63 (42.9%)	45 (43.3%)	18 (41.9%)	
3	38 (25.9%)	23 (22.1%)	15 (34.9%)	
**Ballooning grade (n, %)**				
0	12 (8.2%)	12 (11.5%)	0	**0.001**
1	64 (43.5%)	51 (49.0%)	13 (30.2%)	
2	71 (48.3%)	41 (39.4%)	30 (69.8%)	
**Lobular inflammation grade (n, %)**				
0	6 (4.1%)	6 (5.8%)	0	**<0.001**
1	68 (46.3%)	61 (58.7%)	7 (16.3%)	
2	70 (47.6%)	37 (35.6%)	33 (76.7%)	
3	3 (2.0%)	0	3 (7.0%)	
Histologic NAS	4.82 ± 1.51	4.44 ± 1.46	5.72 ± 1.22	**<0.001**
**Fibrosis stage (n, %)**				
0	28 (19.0%)	28 (26.9%)	0	**<0.001**
1	73 (49.7%)	73 (70.2%)	0	
2	31 (21.1%)	3 (2.9%)	28 (65.1%)	
3	11 (7.5%)	0	11 (25.6%)	
4	4 (2.7%)	0	4 (9.3%)	

Data are medians (IQR), n (%), or means ± SD, unless otherwise specified.

Due to incomplete questionnaires for some subjects, information on smoking history was included in a sample size of only *n* = 137.

The sample size for FAST, Agile 3^+^ was *n* = 146, due to the absence of LSM data for one patient.

Respiratory disease included rhinitis, obstructive sleep apnoea–hypopnoea syndrome, bronchitis, emphysema, or pulmonary nodule.

Bold in column *p*-value represents indicators that are statistically significantly different between the two populations (non-fibrotic MASH and fibrotic MASH).

### Diagnostic performance of eNO

**Figure 1.**
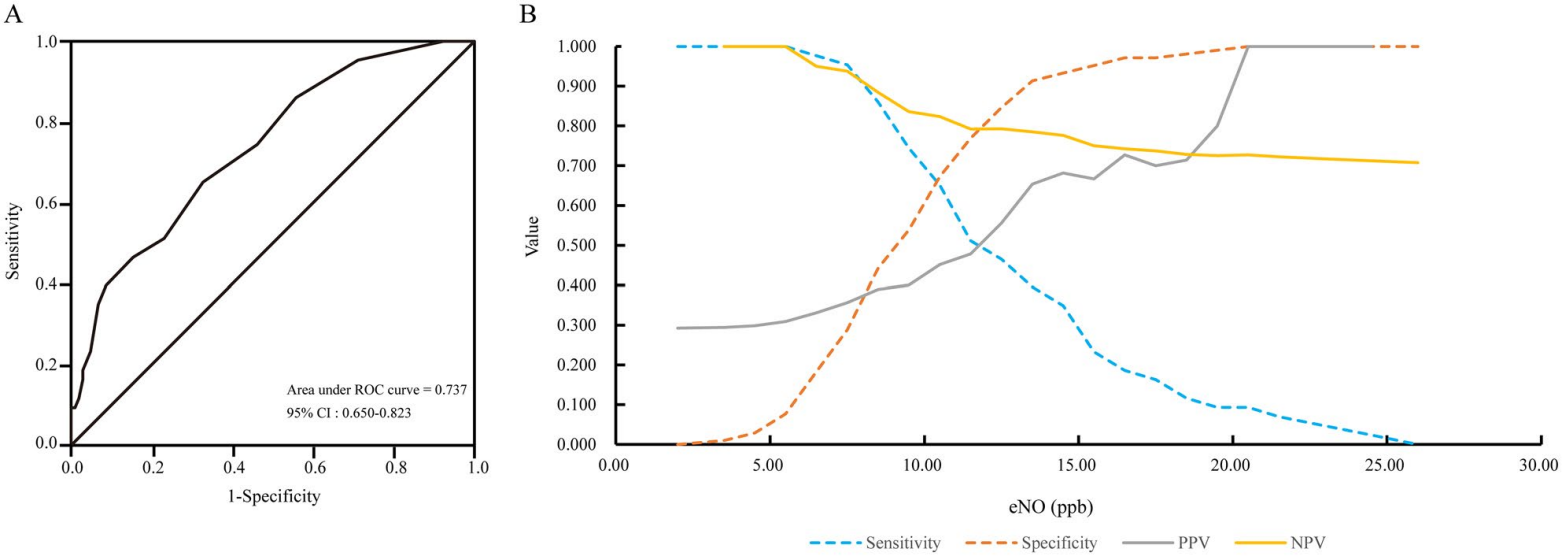
Diagnostic performance of eNO concentration. (A) AUROC of eNO concentration for the non-invasive identification of fibrotic MASH. (B) Sensitivity, specificity, PPV, and NPV versus all possible eNO concentrations. AUROC: area under receiver operation characteristics; PPV: positive predictive value; NPV: negative predictive value.

The AUROC of eNO for the non-invasive diagnosis of fibrotic MASH was 0.737 (95% CI: 0.650–0.823) (Table 2, Figure 1(A)), and Figure 1(B) shows the sensitivity, specificity, PPV, and NPV of eNO for non-invasively diagnosing fibrotic MASH at different cut-off values. Using the dual cut-off approach, the cut-off for sensitivity (>0.85) was 8.5 ppb, and for specificity (>0.9) was 13.5 ppb, which was similar to the efficacy of other published minimally invasive diagnostic indicators using the DeLong test (*p* = 0.820 for FAST; *p* = 0.660 for Agile 3^+^; and *p* = 0.824 for FIB-4, respectively) (Table 2, Supplementary Figure 1).

**Table 2.** Performance of the eNO concentration for the non-invasive detection of fibrotic MASH using dual cut-offs.

Non-invasive test	AUROC (95% CI)	DeLong test p (vs. eNO)	Rule-out zone	Grey zone	Rule-in zone
eNO	0.737 (0.650–0.823)	not applicable	**eNO < 8.5**	**eNO: 8.5–13.5**	**eNO > 13.5**
			n = 52 (35.37%)	n = 69 (46.94%)	n = 26 (17.69%)
			Se = 86.0%		Sp = 91.3%
			Sp = 44.2%		Se = 39.5%
			NPV = 88.5%		PPV = 65.4%
FAST	0.751 (0.656–0.846)	0.820	**FAST < 0.35**	**FAST: 0.35–0.67**	**FAST > 0.67**
			n = 57 (39.04%)	n = 55 (37.67%)	n = 34 (23.29%)
			Se = 81.0%		Sp = 89.4%
			Sp = 47.1%		Se = 57.1%
			NPV = 85.7%		PPV = 69.1%
Agile 3^+^	0.764 (0.670–0.858)	0.660	**Agile 3 < 0.451**	**Agile 3: 0.451–0.679**	**Agile 3 > 0.679**
			n = 122 (83.56%)	n = 11 (7.53%)	n = 13 (8.90%)
			Se = 45.2%		Sp = 99.0%
			Sp = 95.2%		Se = 28.6%
			NPV = 81.1%		PPV = 92.3%
FIB-4	0.721 (0.620–0.821)	0.824	**FIB-4 < 1.3**	**FIB-4: 1.3–2.67**	**FIB-4 > 2.67**
			n = 115 (78.23%)	n = 23 (15.65%)	n = 9 (6.12%)
			Se = 51.2%		Sp = 99.0%
			Sp = 90.4%		Se = 18.6%
			NPV = 81.7%		PPV = 88.9%
LSM	0.763 (0.675–0.851)	0.651	**LSM <9.7 kPa**	**/**	**LSM ≥ 9.7 kPa**
			n = 110 (75.3%)	**/**	n = 36 (24.7%)
			Sp = 85.6%	**/**	Se = 50.0%
			NPV = 80.9%	**/**	PPV = 58.3%

The sample size for FAST, Agile 3^+^, LSM was *n* = 146, due to the absence of LSM data for one patient.

NPV: negative predictive value; PPV: positive predictive value; Se: sensitivity; Sp: specificity.

Bold values represent critical values for each method.

### Sensitivity analyses

Table 3 shows the correlations between eNO content and continuous measurements, with significant associations with LSM (*r* = 0.196, *p* = 0.018), lobular inflammation grade (*r* = 0.208, *p* = 0.012), and fibrosis stage (*r* = 0. 287, *p* < 0.001). Supplementary Figure 2–5 shows characteristics and correlations between NO and liver histology. Sensitivity analysis shows that the AUROC of eNO was not significantly influenced by sex, age, BMI, smoking history, or diabetes (Table 4), with the largest value of AUROC, 0.791, in patients with a BMI of 30 kg/m^2^ or higher. Additionally, to compensate for the absence of external data for validation, 90% of the sample was randomly selected for ROC analysis based on the included sample size, which was conducted 100 times consecutively at random, and all results were between 0.700 and 0.792 (Supplementary Table 1).

**Table 3.** Univariable correlations of eNO with participants’ characteristics.

Parameter	Correlation coefficient	*p*-value
Age (years)	0.150	0.069
BMI (kg/m^2^)	−0.003	0.968
Waist circumference (cm)	0.044	0.599
Smoking history	0.071	0.412
AST (IU/L)	0.029	0.729
ALT (IU/L)	−0.019	0.817
GGT (IU/L)	0.066	0.428
Platelet count (×10^9^/L)	0.069	0.409
LSM (kPa)	0.196	**0.018**
CAP (dB/m)	−0.080	0.335
Steatosis grade	−0.039	0.638
Ballooning grade	0.090	0.280
Lobular inflammation grade	0.208	**0.012**
Histologic NAS	0.101	0.224
Fibrosis stage	0.287	**<0.001**

Pearson’s or Spearman’s correlation analysis was used to assess the univariable correlations between eNO and participants’ characteristics.

Bold represents statistically significant correlation between the corresponding indicator and eNO.

**Table 4.** Accuracy of eNO for diagnosing fibrotic MASH: sensitivity analyses.

	Fibrotic MASH/ No fibrotic MASH	AUROC (95% CI)	Sensitivity	Specificity
Sex				
Female	16/23	0.719 (0.558–0.879)	75.0%	91.3%
Male	27/81	0.769 (0.667–0.817)	92.6%	91.4%
Age (years)				
<40	16/62	0.740 (0.591–0.890)	81.3%	98.4%
≥40	27/42	0.695 (0.573–0.817)	88.9%	81.0%
BMI (kg/m^2^)				
<30	29/66	0.710 (0.603–0.818)	82.8%	87.9%
≥30	14/38	0.791 (0.643–0.940)	92.9%	97.4%
Smoking				
No	28/60	0.739 (0.627–0.850)	78.6%	91.7%
Former + current	12/37	0.775 (0.637–0.912)	100%	91.9%
Diabetes				
No	22/80	0.770 (0.661–0.879)	95.5%	92.5%
Yes	21/24	0.687 (0.532–0.841)	76.2%	87.5%

Due to incomplete questionnaires for some subjects, information on smoking was included in a sample size of only *n* = 137.

Sensitivity at the <8.5 threshold of eNO.

Specificity at the >13.5 threshold of eNO.

### Optimization of eNO diagnostic performance

**Figure 2.**
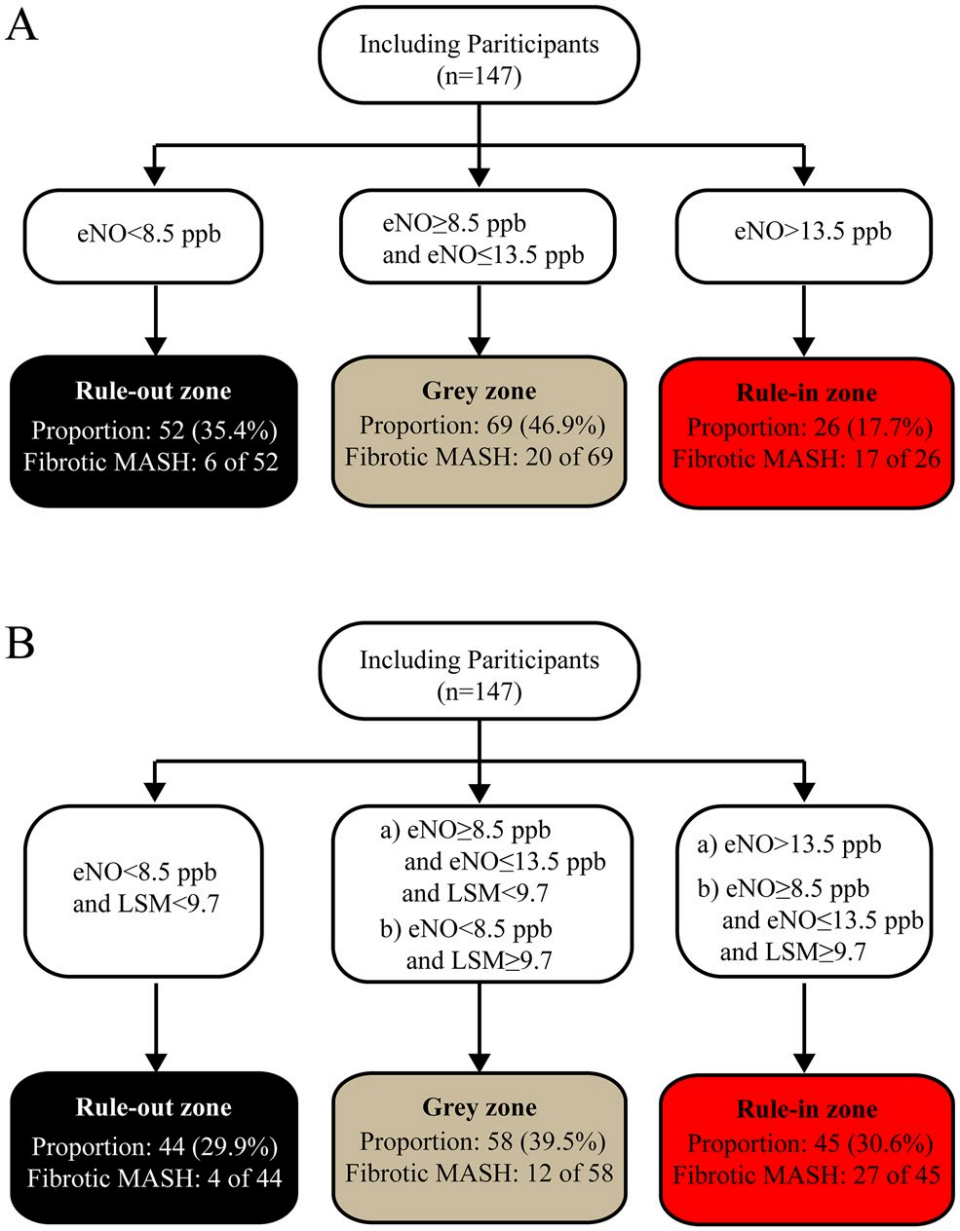
Stratification with a rule-out, “grey zone,” and rule-in zone when applying eNO to identify fibrotic MASH. (A) Using cut-off values of eNO. (B) Using cut-off values of eNO and LSM. LSM: liver stiffness measurement.

**Figure 3.**
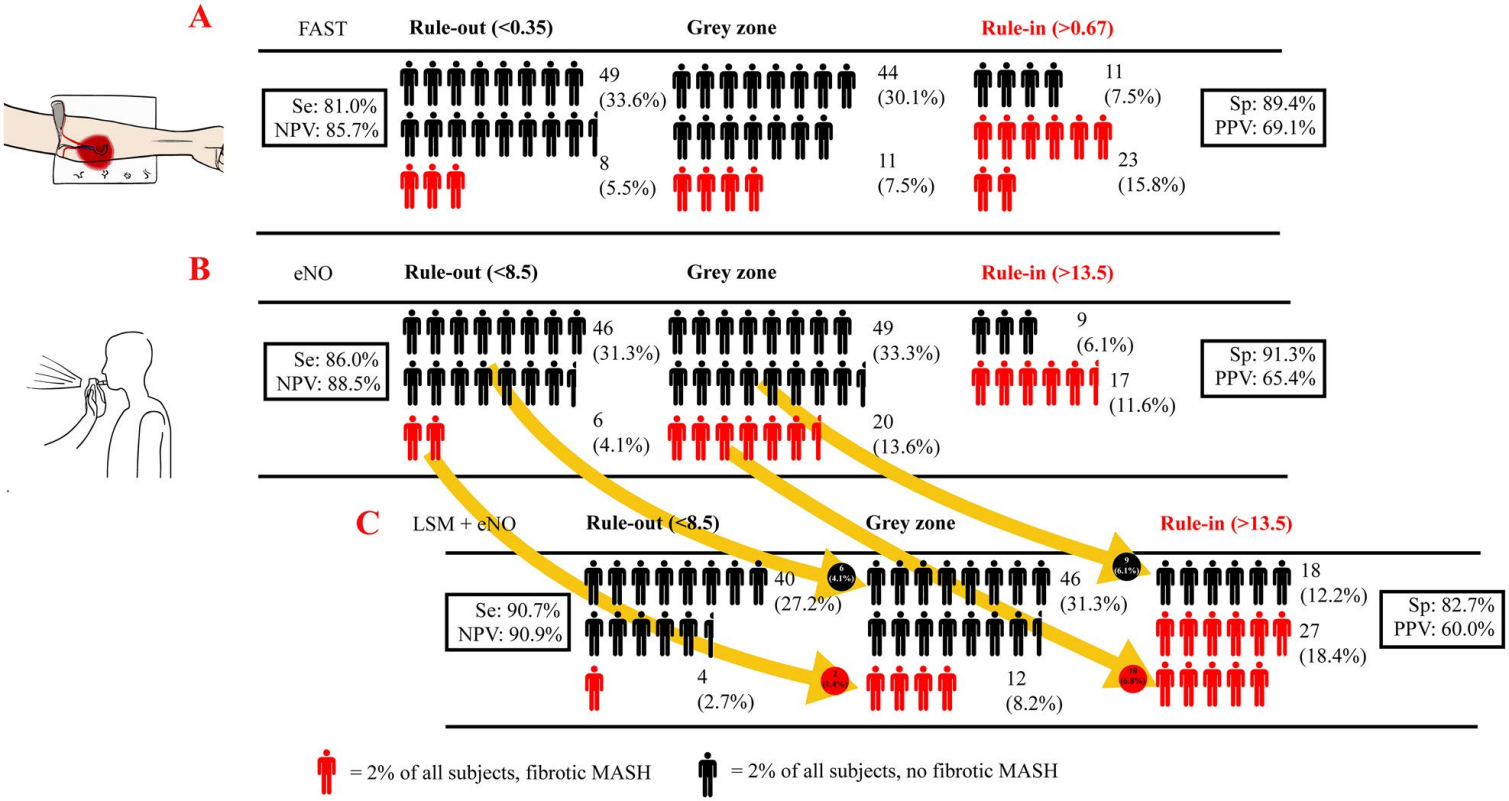
Subjects ruled out and ruled in or remaining in the “grey zone” after: (A) applying the currently recommended FAST cut-offs (0.35, 0.67) or (B) applying the eNO cut-offs (8.5 ppb, 13.5 ppb) obtained herein or (C) applying both the LSM cut-off (9.7 kPa) and eNO cut-offs (8.5 ppb, 13.5 ppb). One full human figure represents 2% of all subjects. Arrows indicate the number (and %) of reclassified patients. For example, when applying LSM as an aid, 10 fibrotic MASH patients (6.8% of the overall patient population) in the “grey zone” were ruled in with their correct diagnosis.

Despite the good diagnostic efficacy of eNO using the dual cut-off approach, the proportion of uncertainty intervals (“grey zone”) was relatively large (69/147, 46.96%). On the other hand, considering the significant correlation between LSM and eNO, as well as the purpose of truly non-invasive diagnosis (rather than minimally invasive), LSM may be a promising indicator in improving the diagnostic efficacy of eNO. Finally, as shown in Figure 2, after introducing LSM as another truncation indicator, using the recommended validate cut-off value of 9.7 kPa [28,29], the proportion of individuals falling into the “grey zone” decreased from 46.9% to 39.5%, similar to the “grey zone” proportion observed with FAST in this same population (Figure 3(A)). The sensitivity further increased from 0.860 to 0.907, and the NPV increased from 0.885 to 0.909, using the dual cut-off approach (Figure 3).

## Discussion

Our novel results show that the eNO-BT is a promising simple test for non-invasively identifying patients with fibrotic MASH. Moreover, the diagnostic performance of the eNO-BT may be improved by adding LSM measurement, and importantly, both of these measurements do not require venesection, which can be problematic in some patients for different reasons.

The choice of fibrotic MASH as the primary outcome of the study was both to enable the inclusion of patients with fibrotic symptoms and to benefit those who may benefit from future anti-inflammatory therapy and to be consistent with the criteria of many current clinical drug trials or diagnostic studies [12,30]. Many available biomarkers have been investigated to diagnose MASH or fibrosis, encompassing clinical parameters, biochemical variables, metabolic factors, and plasma lipids [31–34]. Secondary indicators constructed from the above, such as the FAST [12], Agile 3^+^ [35], and FIB-4 scores [36], have shown good diagnostic accuracy. However, it’s essential to emphasize that these diagnostic tests still fall under the minimally invasive diagnosis category that requires blood tests and are not truly non-invasive.

The overall AUROC of 0.737 for eNO was not significantly different from the FAST score (AUROC = 0.751, DeLong test *p* = 0.820), which was also designed to identify fibrotic MASH. Compared with the other three diagnostic tests, for which cut-off points were derived from published articles [12,35,36], the sensitivity of eNO (Se = 86.0%) at the rule-out threshold was superior to Agile 3^+^ (Se = 45.2%) and FIB-4 (Se = 51.2%). Additionally, eNO achieved a correct diagnosis rate of 80.77% within the rule-in/out area (rule-in zone and rule-out zone) (Supplementary Figure 1).

Fibrotic MASH is characterized by four distinct histopathological lesions (steatosis, lobular inflammation, ballooning, and fibrosis) [37] and subsequent analysis focused on assessing the correlations between eNO and the four histologic features of liver disease (Supplementary Figure 2–5). The positive results on lobular inflammation and fibrosis suggest that the good diagnostic efficacy of eNO for MASH may largely depend on the recognition of the extent of lobular inflammation (Supplementary Figure 2) and fibrosis (Supplementary Figure 5) by eNO.

One of the challenges of screening with eNO is dealing with patients in the “grey zone,” especially considering the current 46.9% proportion in this category in our study (Table 2, Figure 3(B)). Fortunately, by incorporating non-invasive LSM [28,38] data, an indicator obtained by vibration-controlled transient elastography (VCTE), the “grey zone” proportion was reduced to 39% without compromising diagnostic performance (Figures 2 and 3(C)). We also present the diagnostic efficacy of LSM alone in Table 2. The AUROC (0.763 [0.675–0.851]), which was not significantly different from eNO (*p =* 0.651), and the lower sensitivity suggest that the good performance of LSM+eNO is the combined effect of LSM and eNO, rather than the influence of either LSM or eNO in isolation. A dual cut-off approach means the existence of a grey zone is inevitable. As Philip [12] described, decision-making for patients in such an area will be influenced by individual characteristics, threshold proximity, and operator confidence. Repeating the BT at reasonable intervals may be appropriate to refine risk prediction in many situations. In contrast, further alternative methods (even liver biopsy) could be considered in the minority of subjects with diagnostic uncertainty.

While the kappa values for NO and FAST are relatively low at 0.143, they share 43.15% of the same patients in the rule-in area among 146 subjects (Supplementary Table 2). Combined with the different indicators, the discordant data suggested that the diagnostic mechanisms behind both the tests are not identical. eNO has been recognized by authoritative institutions as a reference index for auxiliary diagnosis, differential diagnosis, treatment, and prognosis of respiratory diseases. Guidelines jointly developed by the European Respiratory Society and American Thoracic Society stated that eNO concentration measured by different methods can indicate airway inflammation at different sites [39]. Of the 147 patients with liver disease in our study, 13 had respiratory illness and 49 had a smoking history (former or current). Notably, both indicators were not statistically different in the fibrotic MASH and non-fibrotic MASH participants. Previously, other BTs have been utilized to assess the extent of the disease. For example, the ^13^C-ketoisocaproate-BT and also the ^13^C-methionine breath test have been used to assess mitochondrial dysfunction in NAFLD and to predict higher stages of disease activity [40–42]. Organic volatile organic compounds (*n*-tridecane, 3-methyl-butanonitrile, and 1-propanol) have also been used to diagnose MASH [43].

Our study possesses several important strengths. Firstly, eNO (or eNO + LSM) is not a minimally invasive diagnosis but one of the few truly non-invasive diagnostic tests targeting fibrotic MASH. Our study is the first to test eNO, a respiratory gas, in fatty liver diagnosis. Secondly, the widespread availability of Fibroscan devices based on VCTE technology, the ease of BT, its low cost, and its truly non-invasive nature means this method could easily be used in clinical practice. There is no need for any formulaic calculations (unlike the formulae used in FAST, Agile3, etc.), only the normal value reference range of the accompanying indicator, which can greatly facilitate the understanding of the subjects and the promotion of the method. Thirdly, the classic test for MASH diagnosis is based on liver biopsy, which accounts for a tiny portion of the total liver mass (about 1/50,000) and may be prone to measurement error and poor reproducibility [44] compared with the eNO.

We also acknowledge that there are some limitations to this study. Firstly, the lack of validation in external cohorts remains an unavoidable drawback as few international cohorts of eNO BTs are performed on populations targeting liver disease, even though ROC analysis was performed with 100 randomized replicates in this study to try and circumvent this problem. Secondly, the small sample size in a single ethnic group restricted further analysis and exploration across various population subgroups. One of the complexities in breath analysis is interference from nutritional factors and co-existing pathological respiratory conditions [45]. We may need to study patients with isolated diseases and compare them to patients with multiple diseases to gain insights into the influence of these factors.

In conclusion, the combination of eNO + LSM is a promising, truly innovative, non-invasive combination of two simple tests for identifying fibrotic MASH that we suggest requires further validation in other cohorts and ethnic groups.

## Supplementary Material

Supplemental Material

## Data Availability

The data underlying the results of this study are available upon request because they contain potentially sensitive information. Interested researchers can contact the corresponding author for data access requests via email (zhengmh@wmu.edu.cn).
