# Supplementary material for: Diagnostic accuracy of exhaled nitric oxide for the non-invasive identification of patients with fibrotic metabolic dysfunction-associated steatohepatitis
Source: Ann Med. 2024 Oct 8;56(1):2410408. doi: 10.1080/07853890.2024.2410408 (PMC11463020; doi:10.1080/07853890.2024.2410408)
Supplement: Supplemental Material [file IANN_A_2410408_SM0526.zip › suppl_data/Supplementary Figure caption.docx]

**Supplementary Figure 1**. Number of subjects who were correctly diagnosed based on exhaled nitric oxide. The percentage of correctly diagnosed patients was 63/78 (80.77%) for eNO, 72/91 (79.12%) for FAST, 111/135 (82.2%) for Agile 3^+^, 102/124 (82.25%) for FIB-4.

**Supplementary Figure 2**. Box plot of eNO values in relation to histological characteristic (steatosis grade). The accompanying table provides a pairwise comparison of eNO among different steatosis grades.

**Supplementary Figure 3**. Box plot of eNO values in relation to histological characteristics (ballooning grade). The accompanying table provides a pairwise comparison of eNO among different ballooning grades.

**Supplementary Figure 4**. Box plot of eNO values in relation to histological characteristics (lobular inflammation grade). The accompanying table provides a pairwise comparison of eNO among different lobular inflammation grades.

**Supplementary Figure 5**. Box plot of eNO values in relation to histological characteristics (fibrosis stage). The accompanying table provides a pairwise comparison of eNO among different fibrosis stages.
