# Supplementary material for: Diagnostic accuracy of exhaled nitric oxide for the non-invasive identification of patients with fibrotic metabolic dysfunction-associated steatohepatitis
Source: Ann Med. 2024 Oct 8;56(1):2410408. doi: 10.1080/07853890.2024.2410408 (PMC11463020; doi:10.1080/07853890.2024.2410408)
Supplement: Supplemental Material [file IANN_A_2410408_SM0526.zip › suppl_data/Supplementary tables.docx]

**Table S1**. AUROC curve analysis on 90% of the sample data randomly selected and repeated 100 times.

| **Times** | **No fibrotic MASH (n)** | **Fibrotic MASH (n)** | **Area under ROC** | **95% CI** |
| --- | --- | --- | --- | --- |
| 1 | 91 | 41 | 0.744 | 0.655-0.834 |
| 2 | 93 | 39 | 0.734 | 0.640-0.827 |
| 3 | 97 | 35 | 0.722 | 0.626-0.818 |
| 4 | 94 | 38 | 0.728 | 0.634-0.822 |
| 5 | 94 | 38 | 0.731 | 0.637-0.824 |
| 6 | 94 | 38 | 0.752 | 0.660-0.843 |
| 7 | 95 | 37 | 0.711 | 0.614-0.807 |
| 8 | 93 | 39 | 0.741 | 0.649-0.834 |
| 9 | 96 | 36 | 0.741 | 0.648-0.834 |
| 10 | 92 | 40 | 0.744 | 0.654-0.834 |
| 11 | 93 | 39 | 0.758 | 0.668-0.848 |
| 12 | 92 | 40 | 0.740 | 0.649-0.830 |
| 13 | 93 | 39 | 0.736 | 0.645-0.827 |
| 14 | 93 | 39 | 0.742 | 0.650-0.834 |
| 15 | 92 | 40 | 0.756 | 0.668-0.843 |
| 16 | 93 | 39 | 0.722 | 0.629-0.816 |
| 17 | 93 | 39 | 0.722 | 0.629-0.815 |
| 18 | 93 | 39 | 0.733 | 0.641-0.824 |
| 19 | 96 | 36 | 0.705 | 0.608-0.803 |
| 20 | 92 | 40 | 0.739 | 0.649-0.829 |
| 21 | 95 | 37 | 0.712 | 0.616-0.809 |
| 22 | 92 | 40 | 0.726 | 0.634-0.818 |
| 23 | 91 | 41 | 0.732 | 0.642-0.821 |
| 24 | 95 | 37 | 0.718 | 0.623-0.814 |
| 25 | 93 | 39 | 0.735 | 0.642-0.828 |
| 26 | 94 | 38 | 0.728 | 0.636-0.820 |
| 27 | 97 | 35 | 0.758 | 0.665-0.850 |
| 28 | 91 | 41 | 0.721 | 0.628-0.814 |
| 29 | 95 | 37 | 0.737 | 0.643-0.832 |
| 30 | 91 | 41 | 0.728 | 0.637-0.819 |
| 31 | 95 | 37 | 0.733 | 0.638-0.829 |
| 32 | 94 | 38 | 0.721 | 0.626-0.816 |
| 33 | 93 | 39 | 0.739 | 0.648-0.831 |
| 34 | 94 | 38 | 0.766 | 0.676-0.855 |
| 35 | 94 | 38 | 0.727 | 0.634-0.820 |
| 36 | 95 | 37 | 0.759 | 0.673-0.846 |
| 37 | 93 | 39 | 0.731 | 0.637-0.825 |
| 38 | 93 | 39 | 0.748 | 0.656-0.839 |
| 39 | 92 | 40 | 0.738 | 0.647-0.830 |
| 40 | 94 | 38 | 0.775 | 0.692-0.858 |
| 41 | 93 | 39 | 0.722 | 0.627-0.817 |
| 42 | 92 | 40 | 0.713 | 0.619-0.806 |
| 43 | 94 | 38 | 0.731 | 0.638-0.825 |
| 44 | 92 | 40 | 0.718 | 0.623-0.812 |
| 45 | 94 | 38 | 0.743 | 0.649-0.837 |
| 46 | 92 | 40 | 0.740 | 0.650-0.831 |
| 47 | 94 | 38 | 0.726 | 0.632-0.820 |
| 48 | 95 | 37 | 0.758 | 0.667-0.849 |
| 49 | 93 | 39 | 0.731 | 0.637-0.825 |
| 50 | 95 | 37 | 0.723 | 0.626-0.819 |
| 51 | 92 | 40 | 0.743 | 0.653-0.832 |
| 52 | 91 | 41 | 0.726 | 0.635-0.818 |
| 53 | 96 | 36 | 0.754 | 0.662-0.846 |
| 54 | 94 | 38 | 0.747 | 0.655-0.839 |
| 55 | 93 | 39 | 0.713 | 0.619-0.806 |
| 56 | 92 | 40 | 0.746 | 0.656-0.836 |
| 57 | 96 | 36 | 0.727 | 0.634-0.820 |
| 58 | 94 | 38 | 0.734 | 0.643-0.826 |
| 59 | 92 | 40 | 0.729 | 0.637-0.821 |
| 60 | 93 | 39 | 0.748 | 0.658-0.837 |
| 61 | 94 | 38 | 0.738 | 0.647-0.830 |
| 62 | 93 | 39 | 0.733 | 0.641-0.825 |
| 63 | 91 | 41 | 0.753 | 0.665-0.840 |
| 64 | 96 | 36 | 0.724 | 0.630-0.818 |
| 65 | 95 | 37 | 0.753 | 0.662-0.843 |
| 66 | 94 | 38 | 0.749 | 0.661-0.837 |
| 67 | 94 | 38 | 0.746 | 0.655-0.836 |
| 68 | 96 | 36 | 0.726 | 0.632-0.820 |
| 69 | 92 | 40 | 0.729 | 0.637-0.820 |
| 70 | 93 | 39 | 0.705 | 0.610-0.801 |
| 71 | 91 | 41 | 0.737 | 0.647-0.827 |
| 72 | 93 | 39 | 0.768 | 0.684-0.853 |
| 73 | 94 | 38 | 0.736 | 0.645-0.827 |
| 74 | 92 | 40 | 0.753 | 0.666-0.840 |
| 75 | 90 | 42 | 0.724 | 0.632-0.815 |
| 76 | 90 | 42 | 0.758 | 0.673-0.843 |
| 77 | 94 | 38 | 0.720 | 0.625-0.816 |
| 78 | 93 | 39 | 0.757 | 0.670-0.843 |
| 79 | 93 | 39 | 0.727 | 0.634-0.821 |
| 80 | 95 | 37 | 0.771 | 0.686-0.855 |
| 81 | 93 | 39 | 0.765 | 0.676-0.853 |
| 82 | 94 | 38 | 0.727 | 0.635-0.819 |
| 83 | 94 | 38 | 0.719 | 0.625-0.814 |
| 84 | 93 | 39 | 0.766 | 0.677-0.854 |
| 85 | 92 | 40 | 0.748 | 0.658-0.838 |
| 86 | 95 | 37 | 0.700 | 0.602-0.798 |
| 87 | 93 | 39 | 0.741 | 0.652-0.831 |
| 88 | 94 | 38 | 0.712 | 0.619-0.805 |
| 89 | 91 | 41 | 0.728 | 0.637-0.819 |
| 90 | 93 | 39 | 0.737 | 0.646-0.828 |
| 91 | 93 | 39 | 0.739 | 0.648-0.829 |
| 92 | 92 | 40 | 0.740 | 0.651-0.829 |
| 93 | 93 | 39 | 0.729 | 0.636-0.822 |
| 94 | 90 | 42 | 0.742 | 0.654-0.829 |
| 95 | 95 | 37 | 0.728 | 0.633-0.823 |
| 96 | 94 | 38 | 0.737 | 0.644-0.830 |
| 97 | 93 | 39 | 0.738 | 0.646-0.830 |
| 98 | 93 | 39 | 0.792 | 0.712-0.872 |
| 99 | 92 | 40 | 0.729 | 0.636-0.822 |
| 100 | 93 | 39 | 0.740 | 0.648-0.833 |

**Table S2**. LSM+eNO *and* eNO/FAST agreement [Kappa coefficients and 95% confidence intervals (95% CI)].

|  | **n** | **Kappa (95% CI)** | **Proportion of same patients in rule areas**^†^ |
| --- | --- | --- | --- |
| LSM+eNO and eNO | 147 | 0.719 (0.625-0.813) | 81.63% (120/147) |
| LSM+eNO and FAST | 146 | 0.143 (0.020-0.266) | 43.15% (63/146) |

^†^Proportion of the same patient classified in the same region in both tests (that is, the same patient was classified as being in the rule-in area for both tests, or both were classified as being in the grey area, or both were classified as being in the rule-out area).
