# Supplementary figures and images for: Diagnostic accuracy of exhaled nitric oxide for the non-invasive identification of patients with fibrotic metabolic dysfunction-associated steatohepatitis
Source: Ann Med. 2024 Oct 8;56(1):2410408. doi: 10.1080/07853890.2024.2410408 (PMC11463020; doi:10.1080/07853890.2024.2410408)

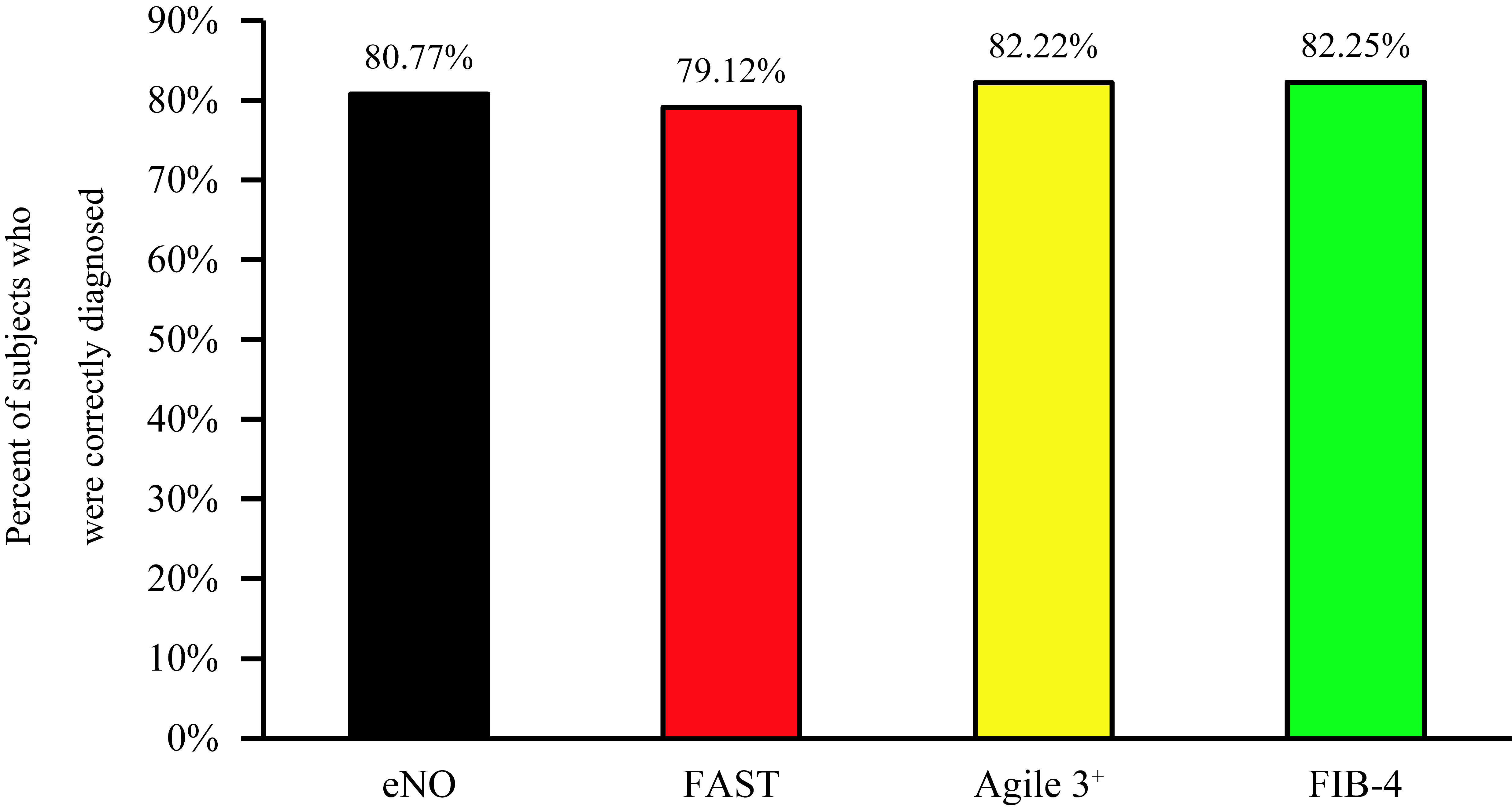

Supplement: Supplemental Material [file IANN_A_2410408_SM0526.zip › suppl_data/Supplementary Figure 1.tif]

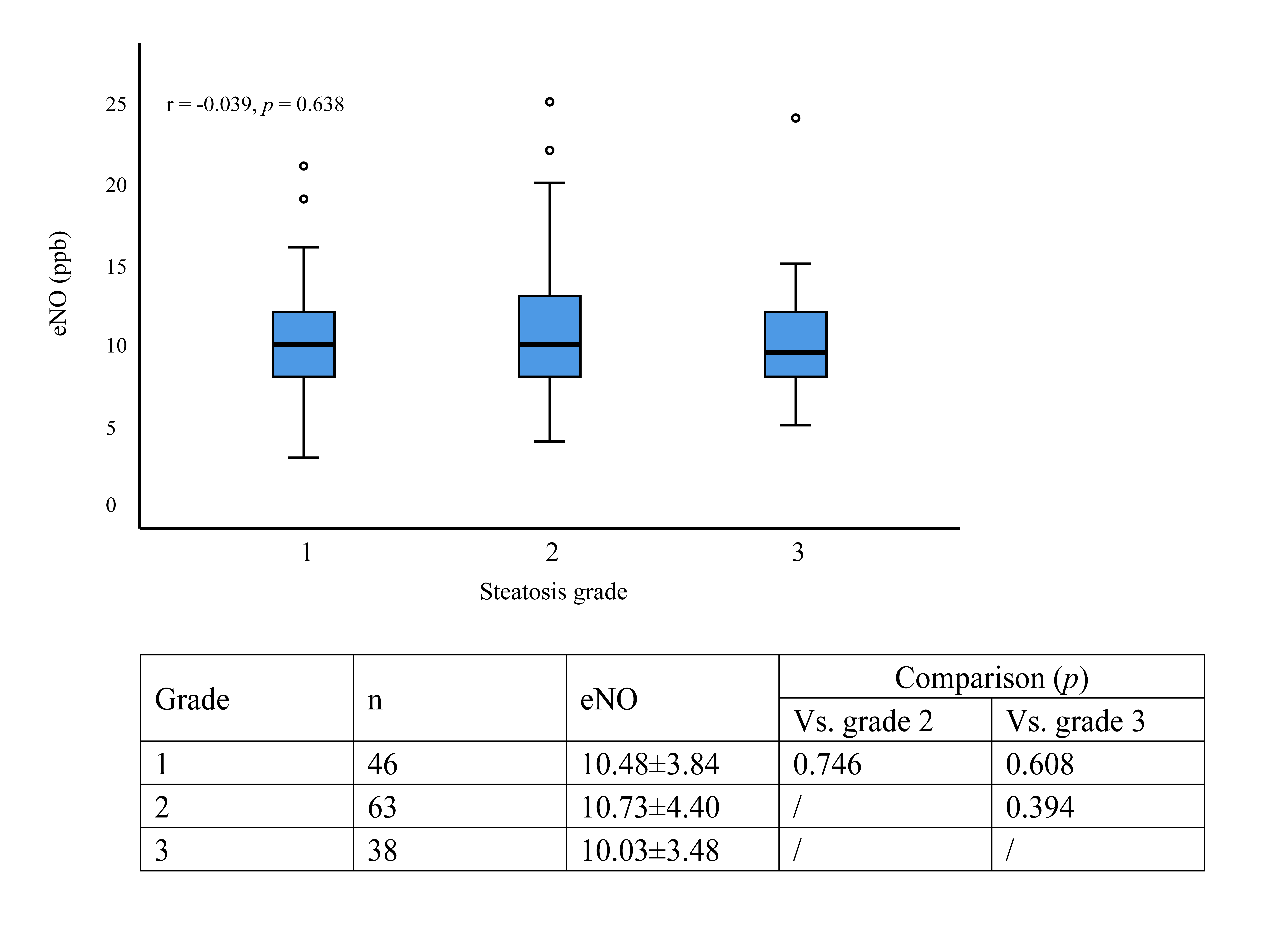

Supplement: Supplemental Material [file IANN_A_2410408_SM0526.zip › suppl_data/Supplementary Figure 2.tif]

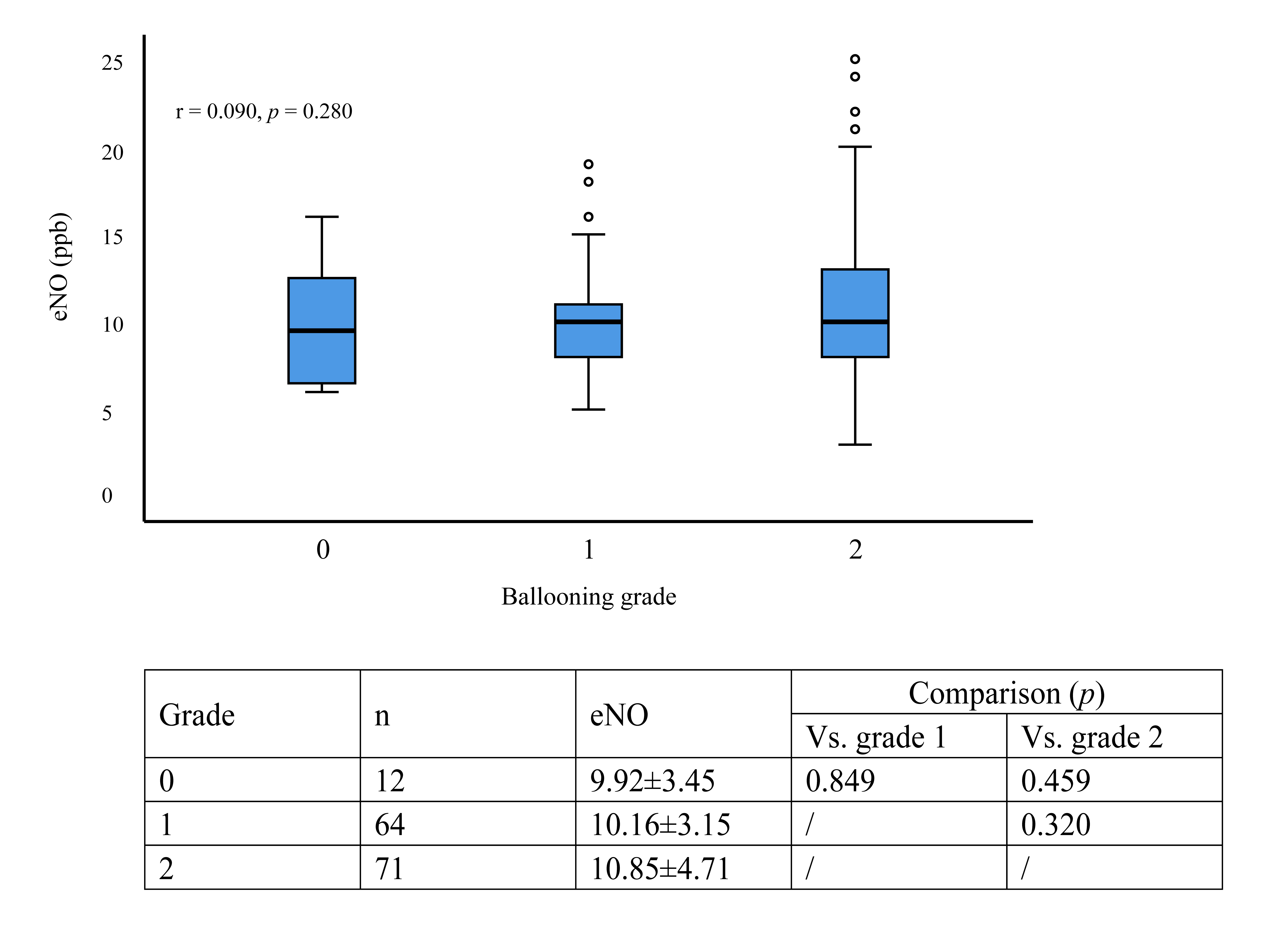

Supplement: Supplemental Material [file IANN_A_2410408_SM0526.zip › suppl_data/Supplementary Figure 3.tif]

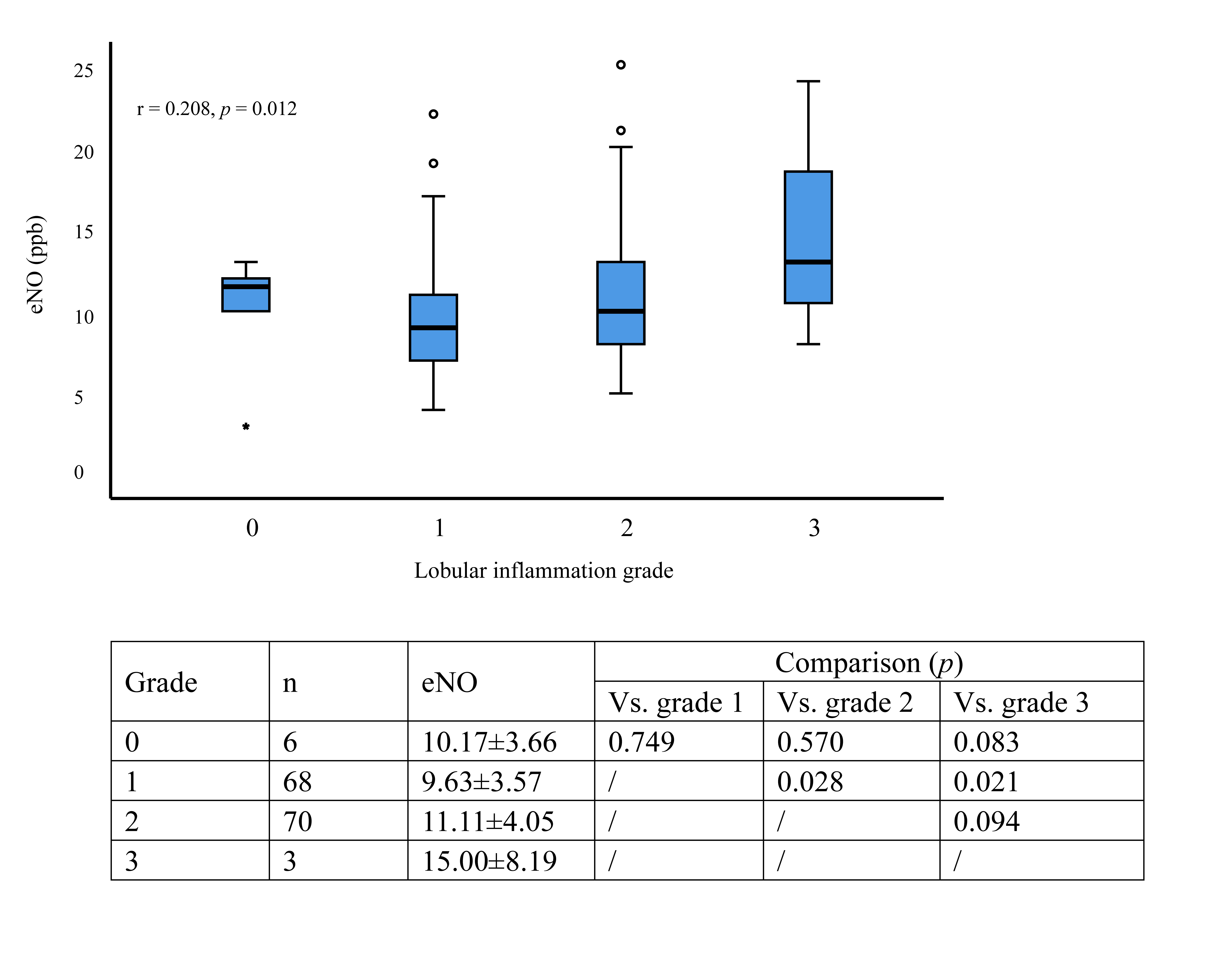

Supplement: Supplemental Material [file IANN_A_2410408_SM0526.zip › suppl_data/Supplementary Figure 4.tif]

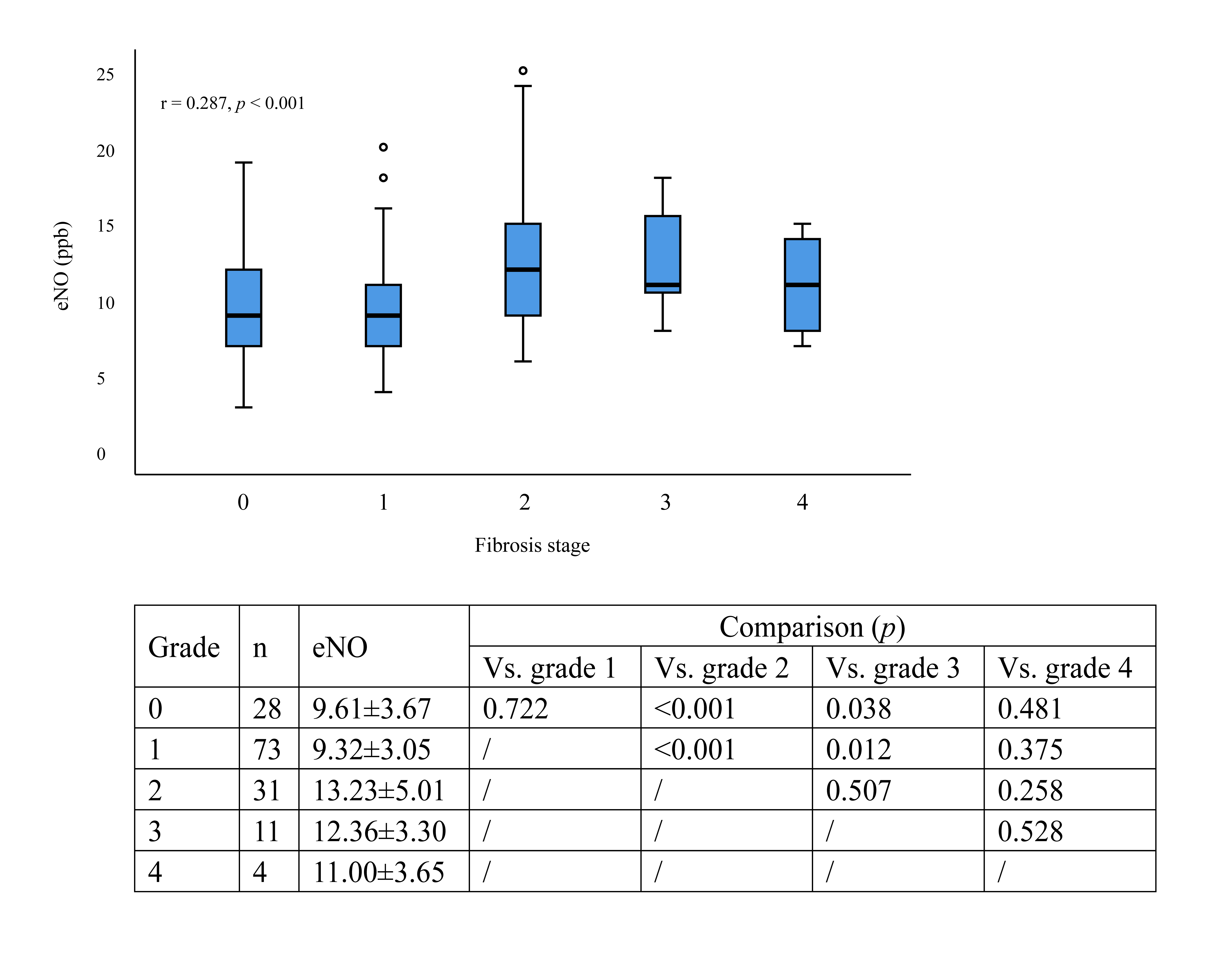

Supplement: Supplemental Material [file IANN_A_2410408_SM0526.zip › suppl_data/Supplementary Figure 5.tif]
